# The pregnant and postpartum stomach

**DOI:** 10.1097/EJA.0000000000002320

**Published:** 2025-11-18

**Authors:** Neel Desai, Jacob Lawson, Francesca Elwen, Ryan Howle

**Affiliations:** From the Department of Anaesthesia, Guy's and St Thomas’ NHS Foundation Trust, London, UK (ND, JL, FE), the Department of Anaesthesia, Rotunda Hospital, Dublin, Ireland (RH), King's College London, London, UK (ND)

## Abstract

For pulmonary aspiration to occur, sufficient gastric content must be present in the stomach, the protective function of the lower oesophageal sphincter has to be overcome and upper airway reflexes must be suppressed or absent. The basal gastric secretion and acidity is not changed in pregnancy. Overall, the evidence indicates that there is a delay in gastric emptying of water in the first trimester compared with the nonpregnant state, but no difference with water or solids in the second and third trimesters of pregnancy. In patients scheduled for elective caesarean delivery, the pre-operative ingestion of a carbohydrate drink, as advocated as part of enhanced recovery after caesarean delivery, leads to no difference in the cross-sectional area of the gastric antrum. Further, the Sip Til Send approach has been found to be noninferior to standard fasting with regard to the cross-sectional area of the gastric antrum and results in beneficial patient reported effects. In women in labour without systemic opioids and without epidural analgesia, gastric emptying was delayed with water and solids, and it was slowed even more with water in the presence of systemic opioids. Importantly, the use of epidural analgesia in labour increased gastric emptying, but not to levels observed in the nonpregnant phase. Clear fluids are likely to be well tolerated in labour, but the intake of solid food remains a concern. In the postpartum period, relative to the nonpregnant state, no difference in gastric emptying with water has been shown. Gastric ultrasound can be performed with the indication, acquisition, interpretation and medical management (I-AIM) framework. The indications for gastric ultrasound in obstetrics include caesarean delivery under general anaesthesia. Once past the first trimester of pregnancy, acquisition involves the semirecumbent and right lateral semirecumbent positions and an awareness of the differences on gastric ultrasound between nonpregnant and pregnant women. The contents of the stomach can be interpreted with qualitative and quantitative evaluation. In qualitative examination, the Perlas system of grading may be used. In quantitative examination, using the threshold of 1.5 ml kg^−1^, measurement of the gastric cross-sectional area as more than 608 mm^2^ in the semirecumbent position, 719 mm^2^ in the right lateral position and 960 mm^2^ in the right lateral semirecumbent position suggest the presence of a full stomach. In elective caesarean delivery with general anaesthesia, the observation of high-risk gastric contents on ultrasound may influence obstetric anaesthetists to postpone the caesarean delivery or reconsider the anaesthetic technique should the indication for general anaesthesia be relative rather than absolute. If no high-risk stomach contents are seen on gastric ultrasound, this may influence obstetric anaesthetists in their choice of induction method: either a modified rapid sequence induction; a standard general anaesthetic induction; full rapid sequence induction and secure the airway with a supraglottic airway rather than tracheal tube. In elective and emergency caesarean deliveries, the information gained from gastric ultrasound might be useful in the setting of failed tracheal intubation.

**KEY POINTS**

- The evidence indicates that there is a delay in gastric emptying with water in the first trimester, compared with the nonpregnant state, but no difference with water or solids in the second and third trimesters of pregnancy.
- Gastric emptying is delayed in labour, although clear fluids are likely to be well tolerated and solids remain a concern.
- Epidural analgesia in labour increases gastric emptying, but not to levels observed in the nonpregnant phase.
- The indications for gastric ultrasound in obstetrics include caesarean delivery under general anaesthesia.
- The use of gastric ultrasound with the indication, acquisition, interpretation and medical management framework is described.
- In the postpartum period, relative to the nonpregnant state, no difference in gastric emptying with water has been shown.

## Introduction

In 1946, Mendelson described 66 cases of pulmonary aspiration in 44 016 pregnant patients, an incidence of 0.15%.^1^ Since then, obstetric anaesthetists have continued to be interested in the pregnant and postpartum stomach and the factors that predispose to the occurrence of pulmonary aspiration. In a national case control and surveillance study conducted in the UK, the incidence of pulmonary aspiration in obstetrics was demonstrated to be 5.2 per 1 000 000 maternity patients.^2^ Further, in 1095 women who had elective or emergency caesarean delivery under general anaesthesia, the incidence of pulmonary aspiration was found to be 0.1% in a prospective observational study.^3^ The risk factors have been reported to be nulliparity and caesarean delivery, particularly under general anaesthesia, with an unadjusted odds ratio of 1.6 and 24.9, respectively.^2^ If failed tracheal intubation occurs in obstetric anaesthesia, then the incidence of pulmonary aspiration has been shown to be 8%.^4^ Should pulmonary aspiration occur, the rate of maternal admission to the ICU, risk of stillbirth and the rate of neonatal decreased Apgar score and admission to the intensive care unit is increased.^2^

It is likely that the concern with regard to the risk of pulmonary aspiration has resulted in the heterogeneity of national and international guidelines with respect to fasting recommendations for caesarean delivery and in labour. For caesarean delivery, the American Society of Anesthesiologists (ASA) and Society of Obstetric Anesthesia and Perinatology (SOAP) advise a fasting interval of 2 h for clear fluids and 6 to 8 h for solids.^5^ In labour, the ASA and SOAP advocate moderate clear fluids in the absence of risk factors for pulmonary aspiration and operative delivery and the avoidance of food,^5^ and the European Society of Anaesthesiology (ESA) recommend clear fluids and the avoidance of food.^6^ Moreover, the National Institute for Health and Care Excellence (NICE) advocate clear fluids such as isotonic drinks and water in labour.^7^ They recommend a light diet should be eaten during labour unless risk factors develop which increase the risk of caesarean delivery, or the mother receives opioids.

Given the worries in relation to the risk of pulmonary aspiration in obstetrics, gastric ultrasound has evolved as a diagnostic and peri-operative point of care tool to evaluate the state of the stomach. It can determine the presence and volume of liquids and solids and differentiate between the empty and full stomach. The aim of this narrative review is to consider the conditions that must be met for pulmonary aspiration to occur; the anatomy, physiology and emptying of the stomach during pregnancy and the postpartum period; and the use and value of gastric ultrasound in clinical decision making.

## Methodology

In order to inform this narrative review, we conducted a search of the literature. The following databases were searched with free text keywords and medical subject headings: Cochrane Central Register of Controlled Trials (CENTRAL); Cumulative Index to Nursing and Allied Health Literature (CINAHL); Elton B Stephens Company (EBSCO) Global Health; Excerpta Medica (EMBASE); Medical Literature Analysis and Retrieval System Online (MEDLINE); Scopus; and World of Science (WoS). The search terms included gastric emptying, gastric motility, stomach, obstetrics and pregnancy. We searched the reference lists of relevant citations for further publications of interest. The quality and relevance of the findings of individual citations were discussed among the authors in a nonstructured manner to reach consensus.

## The occurrence of pulmonary aspiration during pregnancy and postpartum period

For pulmonary aspiration to occur, the following conditions must be fulfilled.^8^ First, fluid content of adequate volume or solid content has to be present in the stomach in order for it to be regurgitated. Second, the protective function of the lower oesophageal sphincter needs to be overcome. The lower oesophageal sphincter is normally closed with a resting pressure of 15 to 25 mmHg above gastric pressure and is under neurohumoral control.^9^ In pregnancy, the pressure of the lower oesophageal sphincter has been demonstrated to decrease in a progressive manner, reaching a nadir of 2.5 mmHg at 36 weeks of gestation and then normalising in the postpartum period.^10^ This reduction in the pressure of the lower oesophageal sphincter is due to the increase in plasma oestrogen and progesterone.^11,12^ Not all studies fully agree with the observation of decreased pressure of the lower oesophageal sphincter in the earlier trimesters of pregnancy. One study found no difference in the pressure of the lower oesophageal sphincter before and after abortion when the patients had been between 14 and 19 weeks of gestation.^13^ However, the response of the lower oesophageal sphincter to hormonal, pharmacological and physiological stimuli was reduced. It has been shown that the intragastric pressure is increased in pregnancy, a likely reflection of the gravid uterus.^14^ Third, the upper airway reflexes which close the laryngeal inlet should be suppressed or absent. The latter two conditions would be met if loss of consciousness resulted from high neuraxial block with regional anaesthesia or if general anaesthesia is required.^15^

## Anatomy and physiology of the stomach

**Fig. 1:**
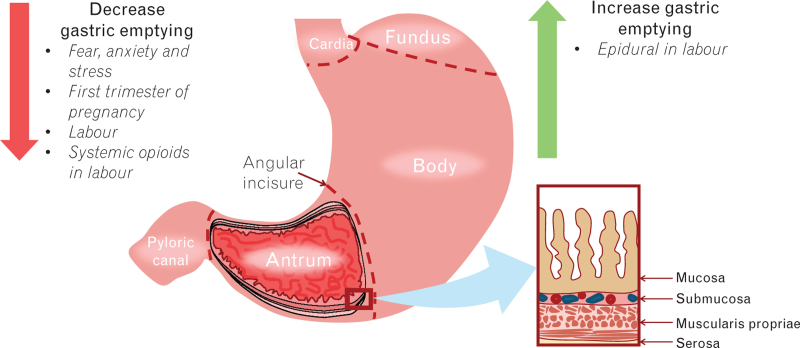
The different anatomical parts of the stomach with a cross-section of the four layers of the gastric antrum on the right.

The stomach can be described as a dilated and distensible muscular bag that is continuous proximally with the oesophagus and distally with the duodenum.^16^ It has a proximal opening, the cardiac orifice and a distal opening, the pyloric orifice. Further, the four main anatomical divisions of the stomach are the cardia, fundus, body and the pylorus (Fig. 1). The cardia is adjacent to the cardiac orifice, the fundus is the part of the stomach that is located superior to the level of the cardiac orifice, the body is the section of the stomach which extends from the cardia to the angular incisure, and the pylorus, composed of a proximal antrum and distal canal, is the part of the stomach that is positioned from the angular incisure to the gastroduodenal junction. Moreover, the wall of the gastric antrum is made up of four layers from luminal to extraluminal, namely the mucosa, submucosa, muscularis propriae and the serosa. Of note, the muscularis mucosae is a sublayer of the mucosa. The physiological functions of the stomach include: the temporary accommodation of ingested fluids and solids; absorption of a limited number of substances such as alcohol and aspirin; mechanical breakdown of food; chemical digestion; regulation of output of chyme (i.e. the semifluid mass of gastric secretions and partially digested food) to the duodenum; secretion of intrinsic factor; and an influence on appetite and satiety.^9^ Importantly, the basal gastric secretion and acidity has been reported to be unchanged in pregnancy.^10^

Factors that decrease and increase gastric emptying have been detailed.

## Gastric emptying

Gastric emptying can be defined as the delivery of the contents of the stomach into the duodenum. It is a complex and multifactorial process that is regulated by hormonal, muscular and neuronal interactions.^17,18^ The excitatory and inhibitory vagal nerves, which are located in the nucleus tractus solitarius, are responsible for the control of gastric emptying.^17^

### Methods of investigating gastric emptying

The historical standard for the investigation of gastric emptying is scintigraphy.^19^ It involves the ingestion of a standardised radiolabelled meal and the measurement of the emitted radiation with a gamma camera, enabling the pattern and rate of gastric emptying to be determined. The standardised meal has a low calorific and fat content that may not be representative of a typical meal and thereby not induce equivalent gastric physiology. Given the radiological nature of scintigraphy, as well as barium x-ray which enables the imaging of barium as it empties from the stomach, these methods are no longer recommended during pregnancy due to the potential harm they may have on the mother and foetus. Other methods to investigate gastric emptying include, in order of frequency, the use of gastric ultrasound, paracetamol absorption, nasogastric sampling with or without dye dilution, epigastric impedance, breath C-octanoic acid analysis,^13^ electrogastrography and the wireless motility capsule.^15^

Gastric ultrasound can measure gastric emptying by the repeated acquisition of sonographic images to observe the contents and volume of the gastric antrum, the dependent region of the stomach.^20^ The cross-sectional area of the gastric antrum is correlated in a linear manner with the total volume of the stomach. In the paracetamol absorption method, the paracetamol is absorbed poorly in the stomach but well in the duodenum and proximal jejunum.^21^ The rate of gastric emptying is therefore the rate limiting step in the delivery of paracetamol into the blood. Given this, the plasma concentration of paracetamol and area under plasma paracetamol curve are proportional and the time to peak paracetamol concentration is inversely proportional to the rate of gastric emptying. The paracetamol absorption method correlates well with scintigraphy, but it does have a number of limitations. First, the absorption of paracetamol may be delayed, at least in part, by its encapsulation with solids if concomitantly administered.^22^ Second, the plasma concentration of paracetamol is influenced by factors such as the volume of distribution and clearance. Should the volume of distribution and clearance increase, as it does with the progression of pregnancy,^23^ then the technique would indicate a delay in gastric emptying even if it were to remain unchanged.

The remaining methods that investigate gastric emptying are now summarised in brief. In the nasogastric sampling method without dye dilution, the volume of the stomach is directly measured. In the nasogastric sampling with dye dilution method, the stomach has dye incrementally added to it and the change in the concentration of dye enables the calculation of its volume.^24^ In the epigastric impedance method, the change in resistance to an alternating current is measured over time.^25^ In the breath ^13^C-octanoic acid analysis method, the octanoic acid is a medium chain fatty acid and, once ingested, is emptied from the stomach and absorbed in the proximal small intestine.^26^ It is then transported to the liver and metabolised to carbon dioxide. The carbon dioxide is subsequently excreted from the lungs where its labelled ^13^C as a specific marker of octanoic acid oxidation is measured. The breath ^13^C-octanoic acid analysis technique correlates well with scintigraphy, but its accuracy can be affected by the presence of malabsorption, liver and lung disease.^19^ In the electrogastrography method, the myoelectrical signals of the stomach are recorded from the skin surface and facilitate the monitoring of gastric activity and motility.^25^ The presence of artifact from movement, however, may result in difficulties with interpretation.^27^ With the wireless motility capsule technique, the pill signals its emptying from the stomach by the sudden change of pH from acidic in the gastric milieu to almost alkaline in the duodenum.^19^

### Limitations of gastric emptying methods

Methods that measure the gastric emptying do not differentiate between the different phases that occur after the ingestion of liquids and/or solids, namely the filling phase when the food is accommodated and the pumping phase where the tonic contractions of the fundus and proximal body and peristaltic contractions of the distal body and proximal antrum of the stomach occur. Given this, subtle changes in the pattern of gastric emptying are not identified by these techniques. Studies which have investigated gastric emptying in the pregnant and postpartum period average the rate of gastric emptying across all patients in their cohort and hence do not account for the effect of comorbidities, such as diabetes, and individual variation.^28^ Importantly, comparisons between these studies should be interpreted with some caution, mainly as they use different methods to measure gastric emptying and the stimulus, depending on the calorific density, chemical composition, osmolality and the volume of the food, may itself have an influence on gastric emptying.^29^ In what follows, it should be recognised that the minimal clinically important difference for the various indices of gastric emptying has not been identified in obstetrics.

### Gastric emptying in the three trimesters of pregnancy

In the first trimester of pregnancy, compared with the nonpregnant phase, gastric emptying has been demonstrated to decrease with water in 93 pregnant patients investigated by the paracetamol absorption method in three observational studies and one randomised controlled trial at moderate risk of bias (Table 1).^30–33^ One observational study at moderate risk of bias, however, did not find the gastric emptying to be reduced with water in 18 pregnant women studied with the same paracetamol absorption method.^18^ Further, another observational study at moderate risk of bias noted no change in the gastric emptying of water between the different trimesters of pregnancy.^32^ No difference was shown by the breath ^13^C-octanoic acid analysis method in the gastric emptying of solids in the first trimester of pregnancy in one observational study at moderate risk of bias that included 24 pregnant women.^34^ In the second trimester of pregnancy, relative to the nonpregnant state, gastric emptying has been found to not be different with water or water and solids in 25 pregnant patients investigated by the paracetamol absorption method in two observational studies at moderate risk of bias.^18,32^ In the third trimester of pregnancy, compared with the nonpregnant state, gastric emptying was not different with either water in 85 pregnant patients investigated by the epigastric impedance, nasogastric sampling with dye dilution, paracetamol absorption or wireless motility capsule methods in four observational studies at moderate risk of bias^18,24,32,35^ or solids in 10 women studied by gastric ultrasound in one observational study at a low risk of bias.^36^ One observational study at moderate risk of bias, however, showed that the gastric emptying was slowed with water and solids in 10 women who were in the third trimester of pregnancy.^37^ Overall, the evidence indicates that there is a delay in the gastric emptying with water in the first trimester, but no difference with water or solids in the second and third trimesters of pregnancy.

**Table 1:** Summary of findings from studies that investigated gastric emptying in the three trimesters of pregnancy, labour and the postpartum period

Study	Group (*n*)	Constituents of groups	Methodology	Drug, liquids and/or solids	Summary of results
Different trimesters of pregnancy					
Clark *et al.*^30^	Pregnant (T1) (30) Nonpregnant (30)	Pregnant and nonpregnant: No premedication (10) Atropine 300 μg (10) Glycopyrrolate 600 μg (10)	Paracetamol absorption	Drug and liquids	In the presence of no premedication, area under plasma paracetamol curve at 1 h decreased in T1 (44.7 ± 12.2 μg ml^−1^ h^−1^) compared to nonpregnant (53.7 ± 16.5 μg ml^−1^ h^−1^) In the context of pregnancy, area under plasma paracetamol curve at 1 h reduced by glycopyrrolate (20.7 ± 10.7 μg ml^−1^ h^−1^) compared to no premedication (44.7 ± 12.2 μg ml^−1^ h^−1^) and atropine (35.8 ± 14.2 μg ml^−1^ h^−1^)
Simpson *et al.*^33^	Pregnant (T1) (28) Nonpregnant (14)	Pregnant: 8 to 11 weeks’ gestation (16) 12 to 14 weeks’ gestation (12)	Paracetamol absorption	Drug and liquids	Area under plasma paracetamol curve at 1 h decreased in 12 to 14 weeks’ pregnant (10.5 ± 5.9 μg ml^−1^ h^−1^) compared to nonpregnant (19.3 ± 7.1 μg ml^−1^ h^−1^) Peak plasma paracetamol concentration reduced in 12 to 14-week pregnant (21.4 ± 7.6 μg ml^−1^) compared to nonpregnant (34.4 ± 16.1 μg ml^−1^) Time to peak plasma paracetamol concentration increased in 12 to 14 weeks’ pregnant (71.9 ± 31.9 min) compared to nonpregnant (45 ± 22.1 min)
Macfie *et al.*^32^	Pregnant (T1–3) (45) Nonpregnant (15)	Pregnant: T1 (15) T2 (15) T3 (15)	Paracetamol absorption	Drug and liquids	Area under plasma paracetamol curve at 1 h decreased in T1 (8.8 ± 1.5 μg ml^−1^ h^−1^) compared to nonpregnant (14 ± 1.5 μg ml^−1^ h^−1^) No difference in area under plasma paracetamol curve at 1 h between T2 or T3 and nonpregnant No difference in peak plasma paracetamol concentration or time to peak plasma paracetamol concentration between T1, T2, T3 and nonpregnant
Levy *et al.*^31^	Pregnant (T1) (20) Nonpregnant (20)	-	Paracetamol absorption	Drug and liquids	Area under plasma paracetamol curve at 1 h decreased in T1 (10.6 ± 5.5 μg ml^−1^ h^−1^) compared to nonpregnant (17.9 ± 8.9 μg ml^−1^ h^−1^) Peak plasma paracetamol concentration reduced in T1 (3.3 ± 7.5 μg ml^−1^) compared to nonpregnant (29.9 ± 11.5 μg ml^−1^) Time to peak plasma paracetamol concentration increased in T1 (69.0 ± 29.0 min) compared to nonpregnant (48.0 ± 28.2 min)
Whitehead *et al.*^18^	Pregnant (T1–3) (64) Postpartum (55) Nonpregnant (32)	Pregnant: T1 (18) T2 (10) T3 (36)	Paracetamol absorption	Drug and liquids	No difference in area under plasma paracetamol curve at 2 h, peak plasma paracetamol concentration or time to peak plasma paracetamol concentration between T1, T2, T3 and nonpregnant
Maes *et al.*^34^	Pregnant (T1) (24) Nonpregnant (43)	Pregnant: No previous hyperemesis gravidarum (10) Recovered hyperemesis gravidarum (14)	Breath ^13^C-octanoic acid analysis	Solids	Gastric half emptying time decreased in T1 recovered from hyperemesis gravidarum (62 ± 20.7 min) compared to T1 without previous hyperemesis (94 ± 21.5 min) No difference in gastric half emptying time between T1 recovered from hyperemesis gravidarum, T1 without previous hyperemesis and nonpregnant
Davison *et al.*^24^	Pregnant (T3) (11) Labour (8) Nonpregnant (11)	-	Nasogastric sampling and dye dilution	Liquids	No difference in gastric half emptying time or volume remaining in stomach at 30 min between T3 and nonpregnant
O'Sullivan *et al.*^35^	Pregnant (T3) (23) Nonpregnant (11)	Pregnant: Magnesium trisilicate (12) Sodium citrate (11) Nonpregnant: Sodium citrate (11)	Radiotelemetry pH pill	Drug and liquids	No difference in gastric half emptying time or pH pill transit time between T3 and nonpregnant with the same antacid No difference in gastric half emptying time between sodium citrate and magnesium trisilicate in T3
Bouvet *et al.*^36^	Pregnant (T3) (10) Labour (20) Nonpregnant (10)	-	Gastric US	Solids	No difference in gastric half emptying time between T3 and nonpregnant
Barboni *et al.*^37^	Pregnant (T3) (10) Nonpregnant (10)	-	Gastric US	Liquids and solids	Change in gastric antral CSA at 10 to 90 min was decreased in T3 (-270 ± 320 mm^2^) compared to nonpregnant (-500 ± 640 mm^2^) Change in gastric antral CSA at 1.5 to 4 h was reduced in T3 (-440 ± 300 mm^2^) compared to nonpregnant (-500 ± 690 mm^2^)
Caesarean delivery					
Popivanov *et al.*^42^	Caesarean delivery (40)	--	Gastric US	Liquids	No solid food detected in gastric antrum and Perlas grade was 0 and 1 in 80%and 20% of patients, respectively, before ingestion Perlas grade at 1.7 h was 0 and 1 in 77.5 and 22.5% of patients, respectively, following ingestion No difference in gastric antral CSA prior to ingestion and subsequent to ingestion at 2 h
Labour					
Influence of different analgesic strategies					
Davison *et al.*^24^	Pregnant (T3) (11) Labour (8) Nonpregnant (11)	Labour: Unspecified analgesia	Nasogastric sampling and dye dilution	Liquids	Gastric volume at 30 min increased in labour (393 ± 126.1 ml) compared to T3 (274.8 ± 115.1 ml) and nonpregnant (186 ± 96.5 ml)
Murphy *et al.*^44^	Labour (80) Caesarean delivery (40)	Labour without opioid: No metoclopramide (20) Metoclopramide (20) Labour with opioid: No metoclopramide (20) Metoclopramide (20) Caesarean delivery: No metoclopramide (20) Metoclopramide (20)	Paracetamol absorption	Drug and liquids	Plasma paracetamol concentration at 1 h decreased in labour with opioid (6.4 ± 8.4 μg ml^−1^) compared to labour without opioid (9.4 ± 6.6 μg ml^−1^), and reduced in labour without opioid (9.4 ± 6.6 μg ml^−1^) relative to caesarean delivery (13.6 ± 6.5 μg ml^−1^) Plasma paracetamol concentration at 1 h decreased in caesarean delivery without metoclopramide (13.6 ± 6.5 μg ml^−1^) compared to caesarean delivery with metoclopramide (17.9 ± 4.7 μg ml^−1^), reduced in labour without metoclopramide (9.4 ± 6.5 μg ml^−1^ relative to labour with metoclopramide (15.9 ± 5.6 μg ml^−1^), and decreased in labour with opioid (6.4 ± 8.4 μg ml^−1^) compared to labour with opioid and metoclopramide (11.5 ± 5.7 μg ml^−1^)
Bouvet *et al.*^36^	Pregnant (T3) (10) Labour (20) Nonpregnant (10)	No epidural (10) Epidural ropivacaine and opioid (10)	Gastric US	Solids	Gastric half emptying time of labour without epidural not calculated Gastric half emptying time increased in labour with epidural ropivacaine and opioid (72 ± 56.3 min) compared to T3 (35 ± 20 min) and nonpregnant (43 ± 23.7 min) Gastric emptying fraction at 1 h decreased in labour without epidural (4 ± 3.7%) or with epidural ropivacaine and opioid (9 ± 11.1%) compared to T3 (34 ± 22.2%) and nonpregnant (27 ± 15.6%) Gastric emptying fraction at 1.5 h reduced in labour without epidural (7 ± 3.7%) compared to labour with epidural ropivacaine and opioid (31 ± 16.3%), T3 (45 ± 18.5%) and nonpregnant (52 ± 11.1%)
Nimmo *et al.*^45^	Labour (46) Postpartum (10)	Labour: No opioid (12) IM diamorphine 10 mg (8) IM diamorphine 10 mg and metoclopramide 10 mg (5) IM pentazocine 60 mg (8) IM pethidine 150 mg (8) IM pethidine 150 mg and metoclopramide 10 mg (5)	Paracetamol absorption	Drug and liquids	Plasma paracetamol concentration decreased at 1 h with pentazocine in labour (2 ± 1.5 μg) compared to no opioid in labour (18.7 ± 4.6 μg) Peak plasma paracetamol concentration reduced with pethidine (14 ± 8.3 μg ml^−1^) and diamorphine in labour (8.6 ± 4.6 μg ml^−1^) compared to no opioid in labour (21 ± 10.3 μg ml^−1^) Time to peak plasma paracetamol increased with pethidine (4 h) and diamorphine in labour (4 h) compared to no opioid in labour (30 min) Inhibitory effect of diamorphine and pethidine on gastric emptying in labour not reversed by metoclopramide
Influence of epidural constituents					
Ewah *et al.*^48^	Labour (36)	Epidural lower concentration bupivacaine alone (4) Epidural lower concentration bupivacaine and higher dose fentanyl (4) Epidural lower concentration bupivacaine and higher dose diamorphine (4) Epidural higher concentration bupivacaine alone (8) Epidural higher concentration bupivacaine and lower dose fentanyl (8) Epidural higher concentration bupivacaine and lower dose diamorphine (8)	Paracetamol absorption	Drug and liquids	Peak plasma paracetamol concentration decreased in labour with lower concentration bupivacaine and higher dose diamorphine (6.4 ± 1.0 μg ml^−1^) compared to labour with lower concentration bupivacaine alone (24.4 ± 4.4 μg ml^−1^) Time to peak plasma paracetamol concentration increased in labour with higher concentration bupivacaine and lower dose fentanyl (75.6 ± 7.4 min) compared to labour with higher concentration bupivacaine alone (45.6 ± 5.6 min), increased in labour with lower concentration bupivacaine and higher dose fentanyl (90 ± 16.6 min) relative to labour with lower concentration bupivacaine alone (45.6 ± 5.6 min), and increased in labour with lower concentration bupivacaine and higher dose diamorphine (257.5 ± 20 min) compared to labour with lower concentration bupivacaine alone (45.6 ± 5.6 min) No difference in peak plasma paracetamol concentration or time to peak plasma paracetamol concentration between all other permutations of concentration of bupivacaine and dose of opioid in labour
Zimmermann *et al.*^49^	Labour (28)	Epidural bupivacaine alone (14) Epidural bupivacaine and opioid (14)	Paracetamol absorption	Liquids	No difference in area under plasma paracetamol curve at 45 min or 1.5 h, peak plasma paracetamol concentration or time to peak plasma paracetamol concentration between labour with epidural bupivacaine alone and labour with epidural bupivacaine and opioid
Postpartum					
Lam *et al.*^50^	Postpartum (100) Nonpregnant (50)	Postpartum: Less than 5 days following neonatal delivery Fasted (50) Nonfasted (50)	Nasogastric sampling	Liquids	No difference in gastric volume between fasted postpartum, nonfasted postpartum and nonpregnant
Scrutton *et al.*^51^	Postpartum (6) Nonpregnant (10)	Postpartum: 18–24 h following neonatal delivery	Gastric US and scintigraphy	Drug, liquids and solids	No difference in rate of gastric emptying between postpartum and nonpregnant

Studies have been placed in the order that they are referred to in text. Differences only reported as significant if *P* < 0.05.

CSA, cross-sectional area; T1, first trimester; T2, second trimester; T3, third trimester.

During in the third trimester of pregnancy, patients may be scheduled for elective caesarean delivery. It is difficult to determine the exact timing of these caesarean deliveries, even when scheduled, and women may often be kept fasted for a prolonged pre-operative period, leading to adverse effects. In response to this problem, they are now encouraged in some centres to drink pre-operative sips of water as part of the Sip Til Send approach until they are called for caesarean delivery. The beneficial effects of this initiative include decreased nausea and vomiting, light headedness and thirst, increased patient comfort and reduced anxiety.^38^ Concerns have been raised, however, with regard to the possible influence of the Sip Til Send approach on the volume of the stomach. The pre-operative protocol of liberal hydration with a maximum of 170 ml of water every h was found in a paired cohort and observational study of 58 patients scheduled for elective caesarean delivery to be noninferior to standard fasting with respect to the cross-sectional area of the gastric antrum.^39^ None of these women had been identified to be at very high risk of pulmonary aspiration or had previous oesophageal or gastric surgery. Further, one institution in the UK has reported no occurrences of pulmonary aspiration following the ingestion of unrestrictive sips of water in over 2000 women before elective caesarean delivery.^40^ The pre-operative intake of a carbohydrate drink has been advocated as part of enhanced recovery after caesarean delivery by the Society for Obstetric Anesthesia and Perinatology.^41^ In an observational study, a carbohydrate drink that had a volume of 400 ml led to no difference in the cross-sectional area of the gastric antrum in 40 patients at two hours and before elective caesarean delivery.^42^ Moreover, the pre-operative ingestion of a carbohydrate drink which had a volume of 500 ml was shown in a randomised controlled trial of 63 women scheduled for elective caesarean delivery to reduce the number of boluses and total dose of phenylephrine subsequent to combined spinal-epidural anaesthesia relative to continued fasting but not water.^43^

### Gastric emptying in labour

Compared with the nonpregnant phase and third trimester of pregnancy, in labour without specified systemic opioids and without epidural analgesia, the gastric emptying of water has been demonstrated to decrease in 48 labouring women investigated by the paracetamol absorption and nasogastric sampling with dye dilution method in one observational study and one randomised controlled trial at moderate risk of bias.^24,44^ No studies, to the knowledge of the authors, have investigated the effect of carbonated fluids on gastric emptying in labour. The gastric emptying of solids was found to be reduced in 10 labouring women who did not have systemic opioids or receive epidural analgesia and were studied with gastric ultrasound in one observational study at low risk of bias.^36^ Metoclopramide increased the gastric emptying in labour without systemic opioids and without epidural analgesia.^44^ Systemic opioids such as intramuscular diamorphine and pethidine, as shown with the paracetamol absorption method in one observational study and one randomised controlled trial at moderate risk of bias, further slowed the gastric emptying with water in labour.^44,45^ The evidence is conflicting in regard to the effect of metoclopramide on the gastric emptying in labour with systemic opioids but without epidural analgesia.^44,45^

In labour with epidural analgesia and in the absence of systemic opioids, compared with the nonpregnant state and third trimester of pregnancy, gastric emptying was demonstrated to decrease with solids in 10 labouring patients investigated by gastric ultrasound in one observational study at low risk of bias.^36^ Importantly, in the same observational study, the gastric emptying was increased with epidural analgesia relative to no epidural analgesia in labour. It is likely that this is reflective of the effect of nociceptive stimuli and pain on the activation of the autonomic nervous systemic and thus gastroduodenal motility and secretion.^46,47^ The constituents of the epidural solution may have an influence on the gastric emptying. No difference was found between epidural lower concentration 0.125% bupivacaine and higher concentration 0.25% bupivacaine in 12 labouring women studied by the paracetamol absorption method in one randomised controlled trial at moderate risk of bias.^48^ The evidence is mixed with respect to the effect of epidural opioids on the gastric emptying in labour.^48,49^ In one randomised controlled trial at moderate risk of bias, gastric emptying was investigated with the paracetamol absorption method in eight labouring patients who had epidural 0.125% bupivacaine with or without fentanyl at a bolus dose of 100 μg.^48^ Fentanyl was found to reduce the gastric emptying. In another randomised controlled trial at moderate risk of bias, 28 women had epidural 0.125% bupivacaine with or without fentanyl at a bolus dose of 50 μg followed by an infusion of bupivacaine and fentanyl.^49^ By the time of blood sampling with the paracetamol absorption method, they had received a cumulative fentanyl dose of 90 μg. No difference in the gastric emptying was shown with fentanyl. Overall, the literature suggests that there is a delay in gastric emptying in labour without epidural analgesia, and in the presence of systemic opioids with epidural analgesia. The use of epidural analgesia in labour does increase the gastric emptying but not back to levels observed in the nonpregnant phase.

### Gastric emptying in the postpartum period

In the postpartum period, no studies have investigated gastric emptying at only the time point when the neonate has been delivered. Relative to the nonpregnant state, no difference in the gastric emptying was shown with water in 106 women at less than five days postpartum by gastric ultrasound and scintigraphy as well as the paracetamol absorption method in two observational studies at moderate risk of bias.^50,51^

## Gastric ultrasound

Gastric ultrasound can be carried out in accordance with the indication, acquisition, interpretation and medical management (I-AIM) framework (Table 2). In order to achieve competence in the qualitative assessment of gastric content using ultrasound, novice gastric sonographers need to conduct 24 and 33 scans in nonpregnant patients to achieve a success rate of 90 and 95%, respectively.^52^ The quantification of gastric volume by novice gastric sonographers, however, is more difficult to learn and accuracy decreases as the ingested volume increases.^53^ Gastric ultrasound, defined as the ability to image the gastric antrum, has a reasonable success rate in the pregnant and postpartum period, and has been found to be 100% in early pregnancy, 90% at greater than 32 weeks of gestation, 88% at more than 36 weeks of gestation and 93% in the postpartum period.^54^ After a learning period of 50 scans, it can be completed in five minutes. Difficulties in ultrasound scanning have been associated with increased BMI in the nonpregnant population.^55^

**Table 2:** The I-AIM framework for the conduct of gastric ultrasound in pregnancy

Indication	Elective caesarean delivery under general anaesthesia	
	If feasible and pragmatic, emergency caesarean delivery under general anaesthesia	
Acquisition	Patient position	If in first trimester of pregnancy or postpartum, in supine and right lateral position If in second or third trimester of pregnancy, in semirecumbent and right lateral semirecumbent position
	Ultrasound device	Curved array and low frequency probe with a frequency of 2 to 5 Hz Conventional abdominal setting
	Ultrasound probe position	In a parasagittal orientation immediately below the xiphisternum and to the right of the midline
	Ultrasound imaging	The following sonographic landmarks should be identified from deep to superficial: vertebral bodies; abdominal aorta; pancreas; left lobe of the liver; and gastric antrum in short axis
Interpretation	Empty	Gastric antrum collapsed and flat, round or ovoid If round or ovoid, it can have a bull's eye or target pattern
	Fluid	Gastric antrum increases in size and its wall thins Presence of anechoic or hypoechoic fluid Presence of hyperechoic dots, due to air bubbles, leading to the starry night appearance
	Solids	Initially, presence of air owing to swallowing of air results in the frosted glass appearance Subsequently, 1 to 2 h later, presence of hyperechoic solids with a heterogenous consistency is observed
	Qualitative evaluation	Perlas system of grading Grade 0 antrum is an empty stomach and Grade 3 antrum refers to a stomach which contains solids Grade 1 antrum indicates that the stomach contains less than or equal to 1.5 ml kg^−1^ in 67% of women Grade 2 antrum suggests that the stomach contains greater than 1.5 ml kg^−1^ in 75% of women If Grade 1 or 2, quantitative evaluation may be performed to quantify the volume of fluid Should quantitative evaluation not be performed, then the Grade 1 antrum should be interpreted as indicating a stomach without high-risk gastric contents and the Grade 2 antrum as suggesting a full stomach with high-risk gastric contents
	Quantitative evaluation	Measure the gastric antral cross-sectional area with the patient in the right lateral or right lateral semirecumbent position Mean of three measurements of the gastric antrum Three formulae available to calculate the gastric volume - - Gastric volume [ml] = 27 + (14.6 x cross-sectional area in the right lateral position [cm^2^]) – (1.28 x age [yrs]) - Gastric volume [ml] = 327.1 + (215.2 x log cross-sectional area in the right lateral position [cm^2^]) - Gastric volume [ml] = (0.24 x cross-sectional area in the right lateral position [mm^2^]) – 54.9 and (0.18 x cross-sectional area in the right lateral position [mm^2^]) + (0.11 x cross-sectional area in the semirecumbent position [mm^2^]) – 62.4 Threshold of fluid volume that differentiates the full stomach from one which is not full is 1.5 ml kg^−1^ Gastric antral cross-sectional area of greater than 608 mm^2^ in the semirecumbent position, 719 mm^2^ in the right lateral position and 960 mm^2^ in the right lateral semirecumbent positions suggest the presence of a full stomach
Medical management	In elective caesarean delivery with general anaesthesia	Consideration of anaesthetic technique of choice If high-risk gastric contents present on gastric ultrasound, consideration of postponing caesarean delivery or reconsidering the anaesthetic technique should the indication for general anaesthesia be relative rather than absolute If high-risk gastric contents not present on gastric ultrasound, consideration of performing modified rapid sequence induction or standard general anaesthetic induction and securing the airway with supraglottic airway In the event of failed tracheal intubation, more information will be available to evaluate whether to wake the woman or proceed with surgery in the event of failed tracheal intubation
	In emergency caesarean delivery with general anaesthesia	In the event of failed tracheal intubation, more information will be available to evaluate whether to wake the woman or proceed with surgery

### Indication

In the third trimester of pregnancy, no difference in gastric emptying has been demonstrated compared with nonpregnant patients.^15^ Consistent with this, two prospective observational studies have found that, after a fast of 2 h for clear fluids and 6 to 8 h for solids, only 3.5 to 5% of 188 patients scheduled for elective caesarean delivery had high-risk gastric contents on ultrasound (Table 3).^56,57^ Some of the evidence, however, is inconsistent with this and of concern. In two prospective observational studies, following a fast of 6 to 7.5 h for clear fluids and 6 to 12.75 h for solids, 38 to 41% of 187 women waiting for elective caesarean delivery still had high-risk gastric contents on ultrasound.^58,59^ The differences in findings between those investigations that evaluate gastric content at a single time point before elective caesarean delivery and those studies which examine gastric emptying over time in the third trimester of pregnancy could be a reflection of the inclusion and exclusion criteria associated with the research on gastric emptying. Only one of the studies that evaluated gastric emptying in the third trimester of pregnancy include women in the lead up to elective caesarean delivery.^32^ It is likely that anxiety, fear, pain or stress are present while waiting for elective caesarean delivery^60^ and factors such as these have been shown to delay gastric emptying.^61,62^ Some of the exclusion criteria in the studies that examined gastric emptying include the presence of obesity,^18^ gastrointestinal disease^18,24,35,36,63^ or multiple pregnancy^18,36^ and the use of any medications^35^ or those which affect gastric motility.^18,32^ Given this, in the opinion of the authors, gastric ultrasound must be considered in all patients in whom elective caesarean delivery has been scheduled under general anaesthesia.

**Table 3:** Overview of the studies that assessed the gastric content in women at a single time point prior to caesarean delivery

Study	Nature of study	Number of patients	Fast for clear fluids (h)	Fast for solids (h)	Presence of high risk gastric contents (%)
Arzola *et al.* 2015^56^	Observational and prospective	103	2	6 for light food such as toast 8 for meat or fatty and fried food	5, all of whom had a calculated fluid volume greater than 1.5 ml/kg
Amaral *et al.* 2019^57^	Observational and prospective	85	2	8	3.5, all of whom had a calculate fluid volume greater than 1.5 ml/kg
Hakak *et al.* 2018^58^	Observational and prospective	46	6	6	37.5, all of whom had a calculated fluid volume more than 1.5 ml/kg
Bouvet *et al.* 2024^59^	Observational and prospective	136	Median 7.5 IQR 3–12	Median 12.75 IQR 11.5–14.5	41, not specified if these patients had a calculated fluid volume more than 1.5 ml/kg or solids

In labour, gastric emptying has been demonstrated to be delayed compared with nonpregnant patients and women in the third trimester in pregnancy.^15^ Clear fluids, however, are likely to be safe in labour as evidenced by a prospective observational study of 62 patients who had epidural analgesia and were allowed to drink clear fluids but ate no solids in labour.^64^ Gastric ultrasound revealed the presence of high-risk gastric contents in 27% of these women at the time of full cervical dilatation, but the duration of fasting for clear fluids was not related to this. Solids remain a concern in labour as evidenced by two studies.^59,65^ In one prospective observational study of 50 patients who had epidural analgesia and were permitted unrestrictive oral intake in labour, 80% of women were found to have high-risk gastric contents when ultrasound was performed at varying times.^65^ In another prospective observational study of 544 patients in spontaneous labour and 288 patients in induced labour, 59 to 68, 55 to 56 and 48 to 51% of women were shown to have high-risk gastric contents on ultrasound subsequent to a fast for solids of between 6 and 8 h, 8 and 12 h and greater than 12 h, respectively.^59^ In view of this, it is the opinion of the authors that gastric ultrasound examination should be considered in all women in whom emergency caesarian delivery is required when feasible and pragmatic.

### Acquisition

Gastric ultrasound can be conducted in the supine and right lateral position for patients who are in the first trimester of pregnancy or postpartum. In those who are in the second and third trimester of pregnancy, it must be performed in the semirecumbent and right lateral semirecumbent position to avoid the effect of supine aortocaval compression^66^ and displace the gravid uterus from the path of the ultrasound.^67^ On placing the woman in the right lateral or right lateral semirecumbent position, the stomach contents gravitate to the gastric antrum, increasing the sensitivity of ultrasound to detect smaller volumes of fluid.^68^ The curved array and low frequency ultrasound probe with a frequency of 2 to 5 Hz and conventional abdominal setting is needed to image the stomach in pregnancy. Some differences on gastric ultrasound are noticeable between pregnant and nonpregnant women. These include the limited space and steep angle between the xiphisternum and uterus, increased depth of scanning, displacement of the stomach in the cephalad direction and to the right, presence of the uterus on ultrasound, and the tachypnoea and hyperdynamic circulation in pregnancy.^69,70^ The ease of gastric ultrasound has been reported to be similar in the semirecumbent and right lateral semirecumbent position in pregnancy.^54^

On ultrasound, the gastric antrum is the portion of the stomach that is most amenable to imaging. It contains less air and is more superficial compared with the body and fundus, and gastric content tends to gravitate towards this region of the stomach. In order to scan the gastric antrum, the ultrasound probe is placed in a parasagittal orientation immediately below the xiphisternum, to the right of the midline above the umbilicus and perpendicular to the skin. It is standard practice for the ultrasound probe to be placed in such a manner that cephalad is on the left of the screen. The following sonographic landmarks should be identified from deep to superficial: the vertebral bodies; long axis of the abdominal aorta; head or neck of the pancreas; inferior margin of the left lobe of the liver; and the gastric antrum in short axis.

### Interpretation

**Fig. 2:**
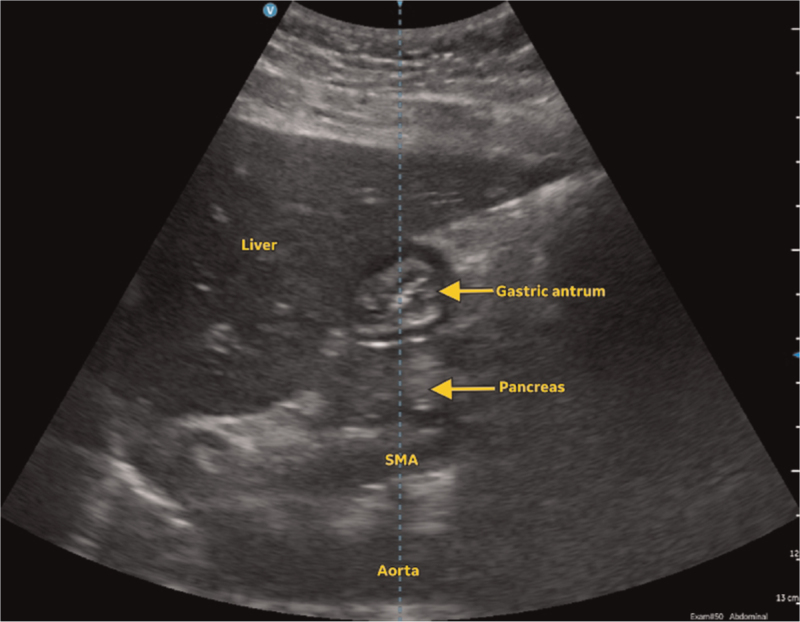
Ultrasound image of the gastric antrum when empty in a patient in the first trimester of pregnancy who has been fasted for 2 h for clear fluids and 6 h for solids.

**Fig. 3:**
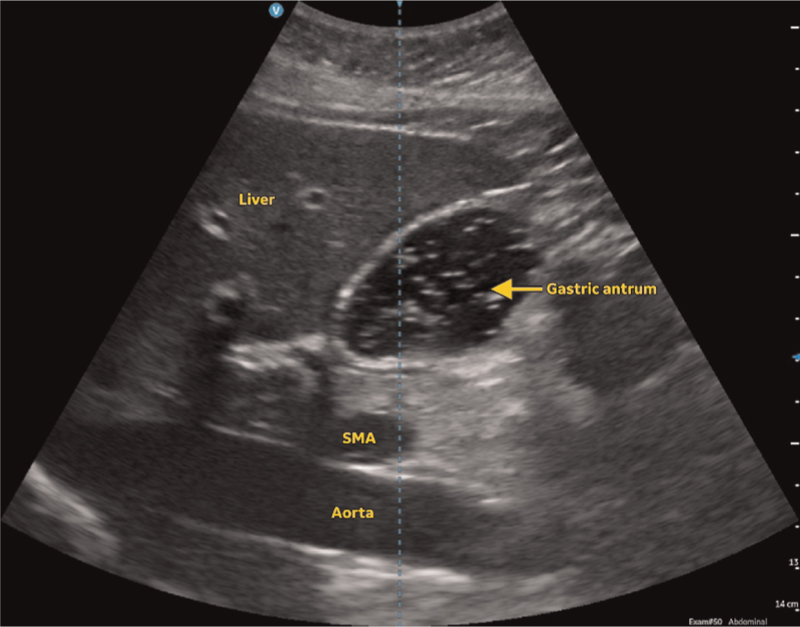
Ultrasound image of the gastric antrum following the ingestion of fluids in a patient in the first trimester of pregnancy.

**Fig. 4:**
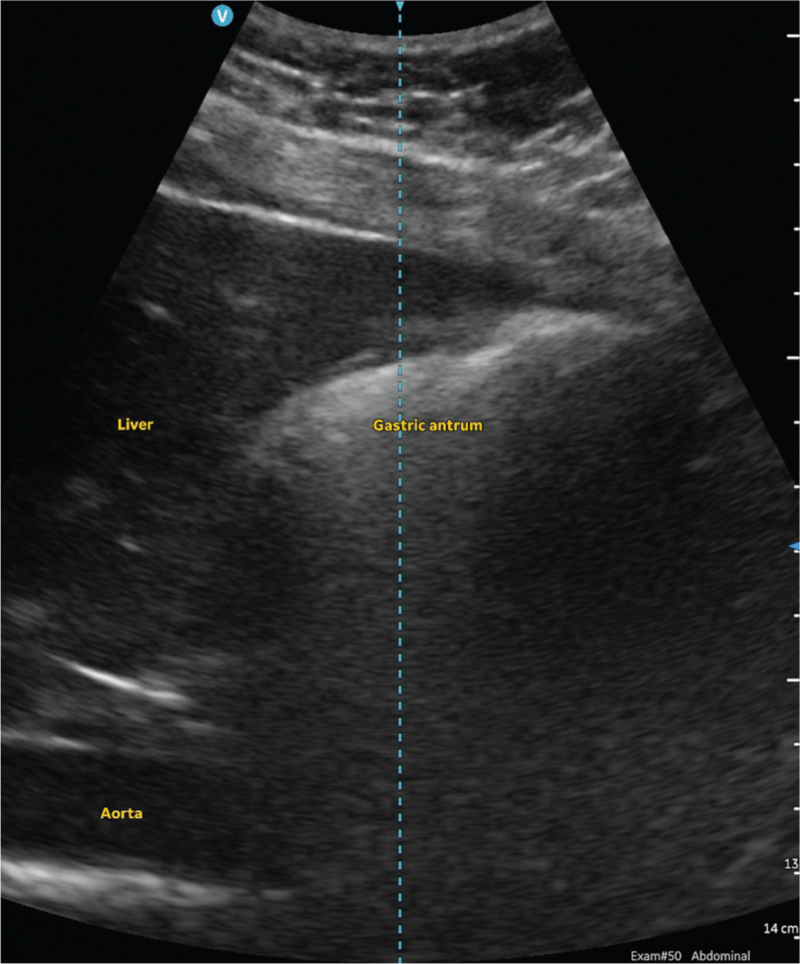
Ultrasound image of the gastric antrum following the ingestion of solids in a patient in the first trimester of pregnancy.

In the empty state, the gastric antrum can appear collapsed and flat, round or ovoid.^20^ If round or ovoid, it has been described as having a bull's eye or target pattern (Fig. 2).^71^ The muscular wall of the stomach, particularly the muscularis propriae layer, is likely to be hypoechoic and prominent with a surrounding hyperechoic outer serosa. It is possible that muscosal folds may be observed in the lumen of the gastric antrum, and these can be differentiated from solids as they will be relatively static rather than moving. Gastric secretions and clear fluids are anechoic or hypoechoic while thicker fluids and milk are more hyperechoic in appearance. Following the ingestion of fluids, the gastric antrum dilates, increasing in size, and its wall becomes thinner (Fig. 3). The process of swallowing clear fluids and carbonated liquids gives rise to the swallowing of air as well, and the presence of air bubbles in the gastric antrum which may be seen as hyperechoic dots, resulting in a starry night appearance. Solids are hyperechoic and have a heterogenous consistency. Following the ingestion of solids, the process of swallowing gives rise to the swallowing of air once again, and the presence of significant air in the gastric antrum then prevents the passage of ultrasound to visualise deeper structures, resulting in the frosted glass appearance (Fig. 4). This air is subsequently absorbed or displaced in the next 1 to 2 h, revealing the presence of the hyperechoic solids with a heterogenous consistency within the distended gastric antrum. It is at this point that peristalsis and the movement of particulate matter may be observed within the distended and thin-walled gastric antrum.

Qualitatively, this is a Grade 0 gastric antrum. The gastric antrum can appear collapsed and flat, round or ovoid. If round or ovoid, as depicted in the ultrasound image, it has been described as having a bull's eye or target pattern. The muscular wall of the stomach, particularly the muscularis propriae layer, is likely to be hypoechoic and prominent with a surrounding hyperechoic outer serosa. SMA, superior mesenteric artery.

Qualitatively, this is a Grade 2 gastric antrum. The gastric antrum dilates, increasing in size, and its wall becomes thinner. The process of swallowing clear fluids and carbonated liquids, however, gives rise to the swallowing of air as well, and the presence of air bubbles in the gastric antrum may be seen as hyperechoic dots, as illustrated in the ultrasound image, resulting in a starry night appearance. SMA, superior mesenteric artery.

Qualitatively, this is a Grade 3 gastric antrum. The process of swallowing gives rise to the swallowing of air once again, and the presence of significant air in the gastric antrum then prevents the passage of ultrasound to visualise deeper structures, as shown in the ultrasound image, resulting in the frosted glass appearance.

**Fig. 5:**
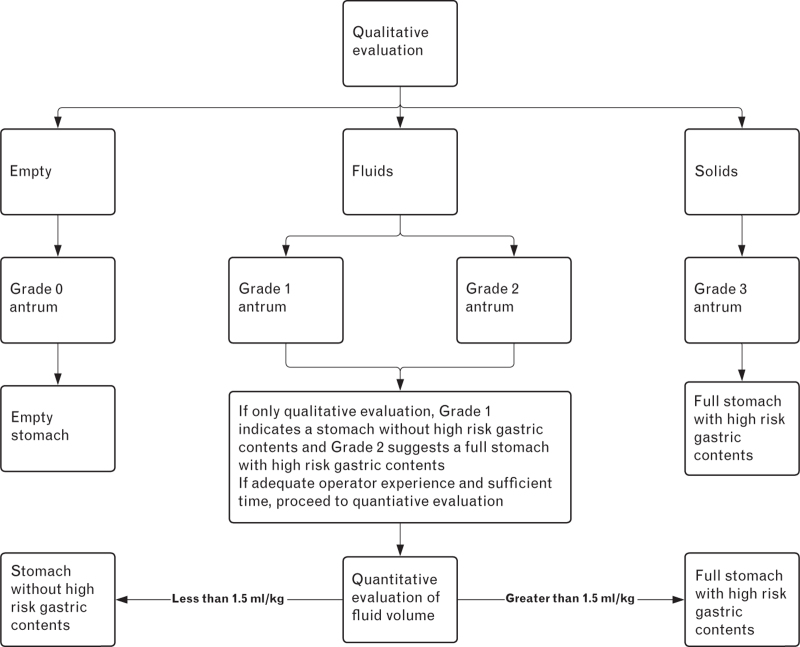
Clinical algorithm for the qualitative and quantitative evaluation of stomach contents.

The contents of the stomach can be characterised with qualitative and quantitative evaluation (Fig. 5). In the qualitative method, the system of grading first described by Perlas for nonpregnant patients may be extrapolated to the pregnant population (Table 4).^72^ With this system of grading, in the semirecumbent and right lateral semirecumbent position, the Grade 0 antrum is empty, Grade 1 and 2 is consistent with the presence of fluids, and the Grade 3 antrum has solids. The Grade 0 antrum is indicative of an empty stomach and the Grade 3 antrum is representative of a full stomach with high-risk gastric contents. No further ultrasound is needed if the Grade 0 or 3 antrum is identified. It is important, however, to evaluate the volume of fluid present in the stomach with either the qualitative or quantitative methods in order to differentiate between the Grade 1 and 2 antrum.

**Table 4:** Summary of the qualitative evaluation of the gastric content with the Perlas system of grading

Grade	Supine or semirecumbent position	Right lateral or right lateral semirecumbent position
0	Empty	Empty
1	Empty	Fluid
2	Fluid	Fluid
3	Solids	Solids

With the qualitative method, the Grade 1 and 2 antrum can be differentiated by assessing the sonographic appearance of the stomach in different patient positions. The Grade 1 antrum is empty in the semirecumbent position and contains fluids in the right lateral semirecumbent position. The Grade 2 antrum has fluids in both the semirecumbent and right lateral semirecumbent position. In the third trimester of pregnancy and with the qualitative method, the Grade 1 antrum was consistent with less than or equal to 1.5 ml kg^−1^ of fluid in 67% of women and the Grade 2 antrum was indicative of greater than 1.5 ml kg^−1^ of fluid in 75% of women.^73^ Overall, the Grade 1 antrum suggested the presence of a stomach without high-risk gastric contents, if this is defined as less than or equal to 1.5 ml kg^−1^ of fluid, and the Grade 2 antrum suggested the presence of a full stomach with high-risk gastric contents, if this is defined as more than 1.5 ml kg^−1^ of fluid, with a sensitivity of 67% and specificity of 87%.

With the quantitative method, the Grade 1 and 2 antrum can be differentiated by calculating the gastric antral cross-sectional area. It is correlated in a linear manner with the total gastric volume.^73–76^ The antral cross-sectional area may be derived by placing the patient in the right lateral or right lateral semirecumbent position and identifying the gastric antrum at the level of the aorta. It is recommended that the gastric antrum is examined at rest between peristaltic contractions when it is at its largest size and the outer serosa should be included within the measurement. The antral cross-sectional area is calculated from the mean of three measurements of the two perpendicular diameters of the elliptical shape of the gastric antrum and through the formula (D1 x D2 x π)/4 or the circumference of the gastric antrum with the calliper tracing tool.

Once the antral cross-sectional area has been calculated, mathematical models can be used to work out the total volume of fluid in the stomach. In the first model, that has been validated in the nonpregnant patient, gastric volume [ml] = 27 + (14.6 x cross-sectional area in the right lateral position [cm^2^]) – (1.28 x age [years]).^77^ In a randomised controlled trial, however, it was found to have a suboptimal statistical linear fit in the third trimester of pregnancy.^75^ In the second model, which was investigated in women in the third trimester of pregnancy, gastric volume [ml] = 327.1 + (215.2 x log cross-sectional area in the right lateral position [cm^2^]).^75^ The coefficient of determination, a statistical measure that represents the proportion of variance for a dependent variable which is explained by an independent variable, was relatively low at 0.44. Further, the limit of agreement between the predicted and ingested volume of fluid was significant at approximately 200 ml. In the third model, which was studied in women in the third trimester of pregnancy, gastric volume [ml] = (0.24 x cross-sectional area in the right lateral position [mm^2^]) – 54.9 and (0.18 x cross-sectional area in the right lateral position [mm^2^]) + (0.11 x cross-sectional area in the semirecumbent position [mm^2^]) – 62.4.^73^ The correlation coefficient, a statistical measure which represents the strength of the linear relationship between two variables, was high at 0.98 when the antral cross-sectional area varied from 300 to 1500 mm^2^, similar to values normally measured in the right lateral semirecumbent position. It is possible that the first model utilised in the nonpregnant population may be appropriate in the earlier stages of pregnancy, and the second and third models might be suitable in the third trimester of pregnancy.

The threshold of fluid volume that should lead to the determination of a full stomach with high-risk gastric contents remains open to debate. In an animal study, the instillation of greater than or equal to 0.4 ml kg^−1^ of acid into the trachea of monkeys resulted in clinical and radiological signs of pneumonitis, and this was more likely to occur once the volume of acid was 0.8 ml kg^−1^ or over.^78^ It has been argued that this potential threshold value of 0.8 ml kg^−1^ of fluid is an underestimation, as it would require the full contents of the stomach to empty into the trachea.^8^ Not only is this considered to be unlikely, but the evidence in humans indicates that higher volumes of up to 1.5 ml kg^−1^ tend to be common in the fasted state and are not related to the incidence of clinically significant pulmonary aspiration.^8,77^ Compared with the threshold of 0.8 ml kg^−1^, setting the threshold for the full stomach at 1.5 ml kg^−1^ of fluid results in increased specificity at the expense of reduced sensitivity. It has been recognised, however, that the use of an arbitrary and single threshold of fluid to differentiate the full stomach from one which is not full is far too simplistic, and does reflect the multifactorial aetiology of pulmonary aspiration.^54^ On the one hand, if we do utilise the threshold of 0.8 ml kg^−1^ of fluid, then an antral cross-sectional area of greater than 505 mm^2^ in the semirecumbent position and 588 mm^2^ in the right lateral position indicates the presence of a full stomach with high-risk gastric contents with a sensitivity of 78 and 74%, respectively, and a specificity of 90 and 83%, respectively.^76^ On the other hand, should we utilise the threshold of 1.5 ml kg^−1^ of fluid, then an antral cross-sectional area of more than 608 mm^2^ in the semirecumbent position, 719 mm^2^ in the right lateral position and 960 mm^2^ in the right lateral semirecumbent position suggests the presence of a full stomach with high-risk gastric contents with a sensitivity of 75, 63 and 80%, respectively, and a specificity of 94, 86 and 67%, respectively.^75,76^ The sensitivity of gastric ultrasound is particularly important in obstetrics in order to reduce the risk of false negatives, that is inadvertently concluding the stomach does not contain high-risk contents when it in fact does.

### Management

Neuraxial anaesthesia is the standard technique for caesarean delivery, but regional anaesthesia can fail and general anaesthesia may be needed or preferable in some circumstances. In the context of elective caesarean delivery, gastric ultrasound can affect decision making in several ways when general anaesthesia is planned. If the fasting history is uncertain, gastric ultrasound may evaluate the fluid and solid status of the stomach. On gastric ultrasound, the observation of high-risk stomach contents may influence obstetric anaesthetists to postpone the caesarean delivery or reconsider the anaesthetic technique should the indication for general anaesthesia be relative rather than absolute. Importantly, no evidence as of yet suggests that further prolongation of the pre-operative fast results in significant emptying should high-risk gastric contents be present on ultrasound. On gastric ultrasound, the visualisation of no high-risk stomach contents may influence obstetric anaesthetists to perform a modified rapid sequence induction or standard general anaesthetic induction rather than full rapid sequence induction and secure the airway with a second generation supraglottic airway rather than tracheal tube. Neither full rapid sequence induction nor intubation with a tracheal tube is without risk.^79^ The use of supraglottic airways has been summarised in a review which included 7722 patients.^80^ None of these decisions should be made on the findings of gastric ultrasound alone and other influencing factors include the experience of the obstetric anaesthetist, presence of comorbidities such as diabetes, obesity and symptomatic reflux, likelihood of difficult tracheal intubation and the potential complexity and difficulty of surgery. In the setting of emergency caesarean delivery, sufficient time may be available to perform gastric ultrasound in category two but not category one surgeries when general anaesthesia is required. In elective and emergency caesarean delivery, the information gained from gastric ultrasound might be useful in the setting of failed tracheal intubation. If failed tracheal intubation occurs, then the multidisciplinary team have a difficult decision to make, that is whether to proceed with caesarean delivery or wake up the woman. The criteria which should be used in the decision-making process have been outlined by the Obstetric Anaesthetists’ Association (OAA) and Difficult Airway Society (DAS).^81^ One of the influencing factors in this situation is the risk of pulmonary aspiration and the information gained from gastric ultrasound would therefore be useful in this regard.

## Conclusion

For pulmonary aspiration to occur, fluid content of adequate volume or solid content must be present in the stomach, the protective function of the lower oesophageal sphincter has to be overcome and upper airway reflexes must be suppressed or absent. The basal gastric secretion and acidity is not changed in pregnancy. Overall, the evidence indicates that there is a delay in gastric emptying with water in the first trimester compared with the nonpregnant state, but no difference with water or solids in the second and third trimesters of pregnancy. In women in labour without systemic opioids and without epidural analgesia, gastric emptying was delayed with water and solids, and it was slowed even more with water in the presence of systemic opioids. Importantly, the use of epidural analgesia in labour increased gastric emptying, but not to levels observed in the nonpregnant state. Gastric ultrasound can be performed with the I-AIM framework. Interestingly, in the setting of elective caesarean delivery, the results of studies which evaluate gastric content at a single time point are conflicting in themselves and inconsistent with studies which examine gastric emptying over time in the third trimester of pregnancy. Clear fluids are likely to be well tolerated in labour, but solids remain a worry. Significant numbers of women have been shown to have high-risk gastric contents on ultrasound. In view of this, the indications for gastric ultrasound in obstetrics include elective or emergency caesarean delivery under general anaesthesia.
